# Elevated serum TIM4 is associated with disease severity and serves as a potential predictive biomarker in chronic hepatitis B

**DOI:** 10.1186/s12879-026-12709-9

**Published:** 2026-02-04

**Authors:** Yong Deng, Juan Mo, Liu Chun, Ning Wang, Jing Cao, Amy Micaela Montufar Mejia, Domenica Camila Montufar Mejia, Muhammad Adil Malik, Dama Faniriantsoa Henrio Marcellin, Xiaowu Li, Zhong Chen

**Affiliations:** 1 Department of Infection and Immunology, The First Hospital of Changsha City (Changsha Hospital Affiliated to Xiangya Medical College, Central South University), Changsha, Hunan 410011 China; 2 Medical Department, The First Hospital of Changsha City (Changsha Hospital Affiliated to Xiangya Medical College, Central South University), Changsha, Hunan 410000 China; 3 Department of Clinical Medicine, The Third Xiangya Hospital, Central South University, Changsha, Hunan 410013 China; 4 Department of Clinical Medicine, Peking University Health Science Center, Peking University, Beijing, 100871 China; 5 Department of Orthopedics, The Third Xiangya Hospital, Central South University, Changsha, Hunan 410013 China; 6 Department of Anatomy and Neurobiology, School of Basic Medical Science, Central South University, Changsha, Hunan 410013 China

**Keywords:** CHB, TIM4, Biomarker, Disease progression, Immunopathogenesis

## Abstract

**Background:**

Chronic hepatitis B (CHB), caused by persistent hepatitis B virus infection, is largely immune-mediated, and biomarkers to identify patients at risk for severe disease are needed.

**Methods:**

In a hospital-based cross-sectional study at the Department of Infectious Disease, The First Hospital of Changsha City (January 2024-October 2025), we enrolled 119 CHB patients (89 non-severe and 30 severe) and 41 healthy controls. Serum T-cell immunoglobulin and mucin domain-containing protein 4 (TIM4) was quantified by enzyme-linked immunosorbent assay (ELISA), and associations with routine laboratory indices and severe CHB were evaluated using correlation analyses, logistic regression, and receiver operating characteristic (ROC) curves.

**Results:**

Serum TIM4 was significantly higher in CHB patients than in controls (*p* < 0.01) and was higher in severe than in non-severe CHB (*p* < 0.05). TIM4 correlated positively with alanine aminotransferase, aspartate aminotransferase, and bilirubin levels (*p* < 0.05) and negatively correlated with platelet count (*p* < 0.01). In both univariate and multivariate (age- and sex-adjusted) analyses, TIM4 was associated with severe CHB (OR = 1.005, *p* = 0.022). ROC analysis demonstrated an AUC of 0.640 (*p* = 0.022).

**Conclusion:**

Serum TIM4 levels are elevated in CHB and are associated with disease severity. TIM4 may serve as a supplementary biomarker for severity assessment; however, further multi-center and longitudinal studies are required to confirm its clinical value.

**Trial registration:**

Not applicable. This is a cross-sectional study and was therefore not registered as a clinical trial.

## Introduction

Chronic hepatitis B (CHB), caused by persistent hepatitis B virus (HBV) infection, remains a major global public health burden and is a primary driver of liver fibrosis, cirrhosis, and hepatocellular carcinoma (HCC) [1–3]. Despite advances in antiviral treatments, predicting the progression from CHB to severe liver diseases such as cirrhosis or HCC remains a significant challenge. The progression of CHB is not only driven directly by viral replication but is also a consequence of immune-mediated liver injury stemming from complex interactions between the host immune system and the virus [1, 4]. A central component of this immunopathology is the dysfunction of the virus-specific T-cell response. Specifically, the functional exhaustion and apoptosis of virus-specific CD8 + T cells are critical factors that prevent viral clearance and sustain persistent hepatic inflammation [5, 6]. Therefore, molecules that modulate T-cell activity are of paramount interest in understanding CHB pathogenesis.

T-cell immunoglobulin and mucin domain-containing protein 4 (TIM4) is a key immunoregulatory member of the TIM family. It is primarily expressed on antigen-presenting cells such as macrophages and dendritic cells. Through interactions with canonical ligands like TIM1 on the surface of apoptotic cells and activated T cells [7, 8], TIM4 is known to modulate T-cell activity and can promote apoptosis, a mechanism implicated in the immune dysregulation observed in contexts of tumor immunity and autoimmune diseases, where it contributes to immune tolerance and suppression [9, 10].

Given this established role of TIM4 in modulating adaptive immune responses and promoting tolerance in chronic inflammatory states, its potential involvement in chronic viral infections characterized by persistent immune activation and subsequent dysfunction is highly plausible. CHB represents a paradigmatic example of such a condition, where persistent HBV antigen exposure drives a uniquely immunosuppressive hepatic microenvironment, leading to T-cell dysfunction, failed viral clearance, and apoptosis [11]. However, within this specific pathophysiological context of CHB, the expression dynamics of TIM4 and its correlation with the spectrum of clinical disease severity remain largely unexplored. Specifically, it is unclear whether circulating TIM4 levels reflect the degree of hepatic immune dysregulation and could thereby serve as a clinically useful biomarker. This gap in knowledge limits our understanding of the immunopathogenesis of CHB and the identification of novel prognostic factors.

In cancer, TIM4 expression has been linked to tumor progression and immune evasion, with studies showing that TIM4-expressing (TIM4+) macrophages contribute to the suppression of anti-tumor immunity [12]. Notably, most mechanistic studies have focused on TIM4 expressed on macrophages, whereas the present study examines circulating TIM4 as a clinical biomarker. In autoimmune diseases, TIM4 has been implicated in regulating immune tolerance and inflammation, with elevated levels found in conditions such as systemic lupus erythematosus (SLE) and rheumatoid arthritis [13, 14]. These findings suggest that TIM4 may play a broader role in immune regulation and disease progression across a range of chronic inflammatory and autoimmune conditions. This highlights its potential as a valuable biomarker not only for liver diseases but also in other immunologically driven conditions.

While TIM4’s role as a T-cell regulator is recognized, its specific expression dynamics within this unique HBV-induced immunosuppressive milieu and its association with determining clinical disease severity are not well defined. This represents a significant knowledge gap. We hypothesize that TIM4, as a mediator of immune inhibition, is upregulated in CHB and that its expression level correlates with the degree of hepatic inflammation and injury, thereby serving as a biomarker for disease severity.

To test this hypothesis, this study was designed with the following objectives: (1) To compare serum TIM4 levels among healthy controls, non-severe CHB patients, and severe CHB patients. (2) To analyze the correlation between TIM4 levels and established markers of liver injury (alanine aminotransferase (ALT), aspartate aminotransferase (AST), total bilirubin (TBIL), and direct bilirubin (DBIL)) and hematological parameters (platelet count (PLT) and complete blood count (white blood cell (WBC), red blood cell (RBC), and hemoglobin (Hb)). (3) To evaluate whether TIM4 is an independent risk factor for severe CHB progression. (4) To assess the predictive value of TIM4 as a potential biomarker for stratifying the risk of severe disease. Investigating TIM4 in this context is crucial for some reasons, including our deep understanding of the immunopathogenesis of CHB, and it may reveal a novel, clinically actionable biomarker for risk stratification and a potential target for future immunomodulatory therapies.

## Materials and methods

### Study design and participants

This was a hospital-based, cross-sectional study conducted at the Department of Infection and Immunity, The First Hospital of Changsha City, which specializes in the diagnosis and treatment of chronic infectious diseases, including CHB. The hospital has extensive experience in managing liver diseases, making it an ideal setting for exploring the role of TIM4 as a biomarker for CHB progression.

A total of 160 subjects were consecutively enrolled between January 2024 and October 2025. The sample size was determined based on statistical power calculations to ensure sufficient data for detecting meaningful differences in TIM4 levels between patient groups. The cohort comprised 119 CHB patients and 41 healthy individuals who underwent routine physical examinations during the same period and served as the control group. Controls were recruited during the same period; age and sex were adjusted in multivariable analyses. This cohort size was considered adequate to provide reliable estimates of TIM4’s association with disease severity, although future studies with larger sample sizes may further strengthen the findings.

#### Patient group and diagnostic criteria

CHB patients were diagnosed according to established guidelines [15]. The diagnostic combinations included criteria based on (1) HBsAg positive for > 6 months; (2) HBsAg positivity with negative HBcAb-IgM; (3) clinical signs of chronic liver disease; (4) persistently or recurrently elevated serum ALT levels, with or without hypoalbuminemia, hyperglobulinemia, or hyperbilirubinemia; (5) liver histology consistent with chronic viral hepatitis; (6) positive HBeAg, anti-HBe, or detectable HBV DNA, after excluding other causes of elevated ALT.

Patients were subsequently stratified into two groups based on disease severity:

- **Non-severe CHB group (*n* = 89)**: Patients who did not meet the criteria for severe CHB.
- **Severe CHB group (*n* = 30)**: Patients meeting the criteria for severe CHB or liver failure, as defined below.

The sample size was estimated a priori using G*Power software (version 3.1.9.7). Based on preliminary data, we assumed a medium effect size (Cohen’s d = 0.5) in serum TIM4 levels between severe and non-severe CHB groups. To achieve 80% power with a two-sided alpha of 0.05 and an allocation ratio of approximately 3:1 (non-severe: severe), a minimum sample size of 27 severe and 81 non-severe CHB patients was required. Our final enrolment of 30 severe and 89 non-severe patients met this requirement.

The criteria for severe or non-severe CHB were adapted from Chinese consensus guidelines on cirrhosis and liver failure and based on established clinical, laboratory, imaging, and histopathological criteria [16, 17]. Notably, these criteria included biochemical markers such as ALT, AST, bilirubin levels, and coagulation indices, as well as evidence of advanced liver disease or hepatic decompensation. HBV DNA testing was available for a subset of patients as part of routine clinical evaluation; however, quantitative HBV DNA data were incomplete and therefore were not included in group comparisons or used as a criterion for severity stratification.

A patient was classified as having severe CHB if they met one of the following combinations in addition to having a positive HBsAg or a clear CHB history: (1) & (2), (1) & (3), or (1) & (4).

Classification as severe CHB required meeting one or more of the following combinations, in addition to a clear CHB history:

1. Combination A: Clinical/Laboratory evidence.

- Criterion 1: Serum HBsAg positivity or definitive CHB history.
- Criterion 2: Presence of one or more clinical complications, including:
- Hypoalbuminemia.
- Elevated ALT/AST.
- Hyperbilirubinemia (with thrombocytopenia and/or leukopenia).
- confirmed esophageal/gastric varices.
- Hepatic encephalopathy.
- Ascites.

2. Combination B: imaging evidence.

- Criterion 1: Serum HBsAg positivity or definitive CHB history.
- Criterion 3: Typical imaging findings of cirrhosis on abdominal ultrasound, CT, or MRI.

3. Combination C: Histopathological evidence.

- Criterion 1: Serum HBsAg positivity or definitive CHB history.
- Criterion 4: Histopathological evidence of diffuse fibrosis and pseudolobule formation.

Alternatively, patients who developed acute-on-chronic failure, meeting the diagnostic criteria outlined in the 2012 Chinese Guideline for the diagnosis and treatment of liver failure, were also included in the.

severe group [16, 17].

##### Exclusion criteria

Patients with co-infections (hepatitis A, C, D, E), non-viral liver diseases (fatty liver disease, alcoholic hepatitis, autoimmune hepatitis, drug-induced liver injury, Wilson’s disease), or any other condition that could independently cause liver damage were excluded.

#### Ethics approval and consent to participate

Written informed consent was obtained from all participants before enrolment. The study protocol (including amendments for extended recruitment through 2025 and TIM4 analysis) was reviewed and approved by the Medical Ethics Committee of The First Hospital of Changsha City [Approval No.: (2021) Ethical Review (Clinical Research) No. 6]. This approval covered the entire study period (January 2024-October 2025) through approved amendments. All procedures were conducted in accordance with the Declaration of Helsinki and its later amendments. Participant confidentiality was maintained throughout the study, and all personal data were anonymized before analysis.

### Data collection and laboratory measurements

#### Baseline data

Demographic information, including age and sex, was recorded for all participants on the first day of admission or examination. Where available, body mass index (BMI) or comorbidity history was also recorded. However, these variables were incomplete and therefore were not included in the baseline comparisons. Additionally, baseline clinical data such as history of liver disease, HBV serological status, and any prior treatment for CHB were also documented.

#### Laboratory parameters

To assess the severity of CHB, several laboratory parameters were measured, including serum TIM4, ALT, AST, TBIL, DBIL, PLT, and complete blood count (WBC, RBC, and hemoglobin (Hb)). These parameters were selected because they are commonly used in clinical practice to monitor liver function and inflammation:

- **ALT and AST**: both are enzymes released into the bloodstream when liver cells are damaged, making them key markers for liver injury and inflammation. Elevated levels are commonly observed in patients with CHB and serve as indicators of hepatocellular damage [18].
- **PLT count**: a well-established marker for assessing the degree of liver cirrhosis and portal hypertension. Thrombocytopenia is a frequent complication in CHB, especially in advanced stages, and is often associated with severe disease and liver failure [19].
- **TBIL and DBIL**: elevated bilirubin levels reflect impaired liver function, specifically bile excretion, which can result from liver injury and cirrhosis. Bilirubin levels are frequently used to evaluate the severity of liver dysfunction [20].

These parameters were chosen for their clinical relevance and their established role in the diagnosis and monitoring of CHB progression. Their inclusion helps contextualize the role of TIM4 as a potential biomarker of immune dysregulation and liver injury.

#### Blood sample processing and assays

Fasting venous blood (5 mL) was collected from each participant on the first day. Serum was separated.

by centrifugation at 2000 × g for 10 min at room temperature, aliquoted, and immediately stored at.

-80 °C until batch analysis.

- **Serum TIM4** concentration was quantified using a commercially available Human TIM4 Enzyme-Linked Immunosorbent Assay (ELISA) kit (Cat # EK-053-96, LOT: 202309 A; Wuhan Fine Biotech Co., Wuhan, China) according to the manufacturer’s instructions. Absorbance was measured at 450 nm using a fully automated ELISA analyzer (Thermo Fisher Scientific, USA).
- **Routine biochemical and hematological parameters**, including ALT, AST, TBIL, DBIL, blood urea nitrogen (BUN), and serum creatinine (SCr), were measured using a fully automated biochemical analyzer (Roche, Switzerland). Complete blood count parameters, including WBC, RBC, Hb, and PLT, were determined using a fully automated hematology analyzer (Sysmex, Japan).

### Statistical analysis

All statistical analyses were performed using SPSS software (version 25.0, IBM Corp.) and R software (version 4.1.0, R Foundation for Statistical Computing) for specific analyses, such as Receiver Operating Characteristic (ROC) curve analysis and logistic regression. Categorical variables are presented as numbers (n) and percentages (%), and comparisons among groups were made using the chi-square (_*χ*_^2^) test. For continuous variables, normality was assessed using the Shapiro-Wilk test. Normally distributed data are expressed as mean ± standard deviation (SD) and were compared among the three groups using one-way analysis of variance (ANOVA), followed by the Bonferroni-adjusted pairwise comparisons. Non-normally distributed data are presented as median (interquartile range, IQR) and were compared using the Kruskal-Wallis H test, followed by the Bonferroni-adjusted Mann-Whitney U test for pairwise comparisons. A two-tailed p-value of < 0.05 was considered statistically significant, and *p* < 0.01 was considered highly significant.

Missing data were handled using listwise deletion, where subjects with missing values for key variables were excluded from the analysis. In cases where missing data were minimal (i.e., < 5% of the dataset), no imputation was performed, as this did not significantly affect the validity of the statistical results. Sensitivity analyses were conducted to assess the impact of missing data on the study’s conclusions.

To adjust for the potential inflation of Type I errors due to multiple comparisons, the Bonferroni correction was applied for multiple comparisons during pairwise post-hoc testing following significant ANOVA or Kruskal-Wallis results. The adjusted significance level was calculated as ⍺/n, where n is the number of pairwise comparisons between the three groups (*n* = 3), yielding a corrected alpha of 0.0167. This adjustment was used to control the family-wise error rate, ensuring that the statistical significance observed is not due to random chance.

Bivariate correlation analysis (Pearson or Spearman, as appropriate based on data distribution) was used to examine the relationships between serum TIM4 levels and other laboratory parameters. Correlation coefficients (r) > 0 indicated a positive correlation, *r* < 0 indicated a negative correlation, and the absolute value of r reflected the strength of the association.

To identify factors associated with severe CHB, univariate binary logistic regression analysis was performed. The dependent variable was disease status (non-severe = 0, severe = 1), and the independent variables were the measured laboratory parameters. Results are presented as odds ratios (OR) with 95% confidence intervals (CI).

Finally, to determine the cutoff value for TIM4, we performed an ROC curve analysis. This analysis is widely used to evaluate the diagnostic ability of a biomarker and to identify the optimal threshold for distinguishing between different disease states. The area under the ROC curve (AUC) was calculated, and the optimal cutoff value for TIM4 was determined by maximizing Youden’s index (sensitivity + specificity − 1), a method that maximizes the sum of sensitivity and specificity. This ensures that the selected threshold provides the best balance for identifying patients at risk for severe disease progression. The selected cutoff value of 447.68 pg/mL provided a sensitivity of 63.3% and specificity of 62.9%, indicating a moderate ability to predict severe CHB. For combined biomarker analysis, a multivariable logistic regression model including TIM4, ALT, and AST was constructed, and predicted probabilities from this model were used to generate the receiver operating characteristic (ROC) curve.

## Results

### Baseline characteristics of the study cohort

A total of 160 subjects were included in the final analysis: 41 healthy controls, 89 patients with non-severe CHB, and 30 patients with severe CHB. The cohort comprised 105 (65.6%) males and 55 (34.4%) females, with a mean age of 44.14 ± 14.33 years.

As shown in Table 1, both CHB patient groups were significantly older than the healthy control groups (*p* < 0.01), and patients in the severe CHB group were older than those in the non-severe group (*p* < 0.05). The proportion of male participants was significantly higher in both CHB groups compared to the control group (*p* < 0.01), but did not differ significantly between the non-severe and severe CHB groups (*p* > 0.05).

**Table 1:** Baseline demographic characteristics of the study participants

Characteristics	Healthy Controls (*n* = 41)	Non-severe CHB (*n* = 89)	Severe CHB (*n* = 30)	*p*-value
Age, years (mean ± SD)	35.29 ± 7.79	48.53 ± 12.22 **	56.23 ± 12.24^**^†	< 0.001
Male/Female, n (%)	12 (29.3)/29 (70.7)	68 (76.4)/21 (23.6) **	25 (83.3%) /5 (16.7) **	< 0.001

** *p* < 0.01 vs. healthy control group; †*p* < 0.05 vs. non-severe CHB group

### Comparison of laboratory parameters

**Fig. 1:**
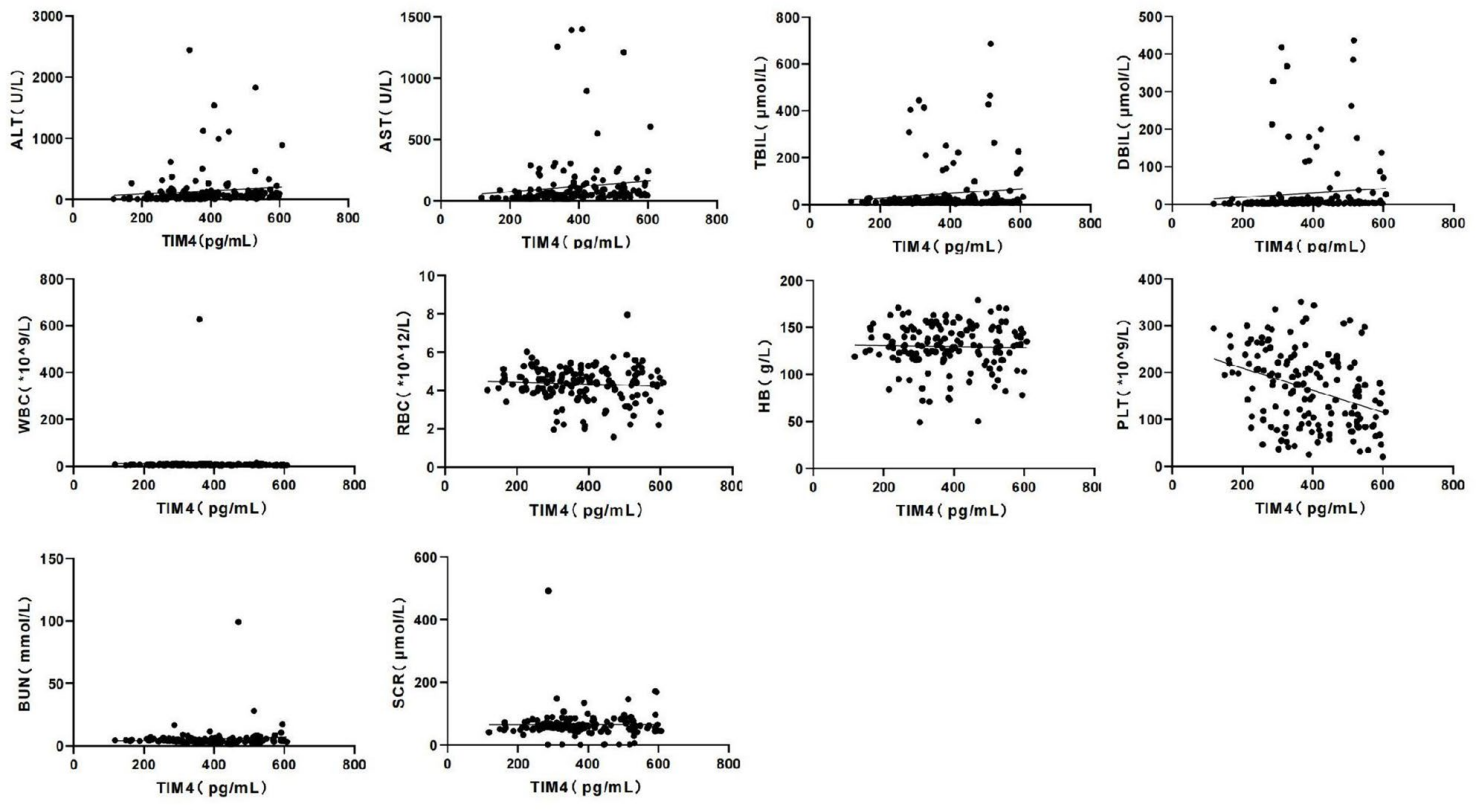
Scatter plots demonstrating correlation analysis between serum TIM4 and laboratory parameters. Parameters analyzed include liver function markers (ALT, AST, TBIL, and DBIL), biochemistry markers (BUN and SCr), and hematological markers (WBC, RBC, Hb, and PLT). Each panel displays the correlation coefficient (r) and its statistical significance (p-values) from bivariate analysis. Solid regression lines indicate statistically significant correlations (*p* < 0.05), whereas dashed lines indicate non-significant associations. ALT (*r* = 0.33, *p* < 0.01), AST (*r* = 0.35, *p* < 0.01), TBIL (*r* = 0.16, *p* = 0.04), DBIL (*r* = 0.29, *p* < 0.01), PLT (*r* = -0.36, *p* < 0.01), WBC (*r* = -0.08, *p* = 0.31), RBC (*r* = -0.07, *p* = 0.38), Hb (*r* = -0.03, *p* = 0.73), BUN (*r* = -0.04, *p* = 0.65), SCr (*r* = 0.02, *p* = 0.80)

The comparison of laboratory parameters across the three groups is summarized in Table 2. Serum TIM4 levels were significantly higher in both CHB groups compared to the healthy control group (*p* < 0.01), with the highest levels observed in the severe CHB groups (*p* < 0.05 vs. non-severe). This progressive increase in TIM4 levels from healthy controls to non-severe and severe CHB patients suggested that TIM4 may reflect disease severity (Fig. 1). In addition, TIM4 levels showed significant positive correlations with traditional markers of liver injury and inflammation, including ALT, AST, TBIL, and DBIL (*p* < 0.05, Fig. 1), reinforcing its potential as a biomarker for immune dysregulation and liver injury in CHB.

Regarding other laboratory indices, several hematological parameters, including WBC, RBC, and PLT, were significantly lower in CHB patients compared to healthy control groups (*p* < 0.05). In contrast, Hb level did not differ significantly between the overall CHB cohort and the control group (*p* > 0.05). When comparing the two patient groups, only TBIL was significantly higher in the severe CHB group (*p* < 0.05). As summarized in Table 2, no significant differences were observed among the three groups for Hb, BUN, or SCr levels (*p* > 0.05).

**Table 2:** Comparison of laboratory parameters among the study groups

Characteristics	Healthy Controls (*n* = 41)	Non-severe CHB (*n* = 89)	Severe CHB (*n* = 30)
TIM4 (pg/mL)	286.07 ± 94.33	411.27 ± 107.93^**^	464.53 ± 102.12^**^†
ALT (U/L)	14.70 (9.70, 22.95)	74.20 (39.90, 129.40) ^**^	98.30 (44.40, 145.90) ^**^
AST (U/L)	22.10 (20.50, 24.45)	62.30 (42.80, 117.60) ^**^	92.00 (55.20, 236.70) ^**^
TBIL (µmol/L)	10.80 (9.80, 14.70)	18.50 (10.10, 32.10) ^*^	22.00 (12.40, 149.70) ^**^†
DBIL (µmol/L)	2.20 (1.80, 2.90)	6.30 (4.30, 16.50) ^**^	9.90 (3.80, 87.40) ^**^
WBC (*10^9/L)	5.97 (4.70, 6.56)	4.82 (3.21, 6.42) ^**^	4.75 (3.91, 6.03) ^**^
RBC (*10^12/L)	4.67 ± 0.57	4.27 ± 0.96^*^	4.04 ± 1.06^*^
Hb (g/L)	133.88 ± 19.58	128.66 ± 24.62	127.52 ± 28.87
PLT (*10^9/L)	243.05 ± 43.67	141.67 ± 75.77^**^	122.83 ± 57.97^**^
BUN (mmol/L)	4.39 (3.79, 4.90)	4.14 (3.33, 6.04)	4.44 (2.98, 5.11)
SCr (µmol/L)	53.80 (47.75, 67.20)	60.80 (50.20, 72.50)	63.10 (48.80, 87.90)

**TIM4** levels were significantly elevated in both CHB groups compared to healthy controls (** *p* < 0.01 vs. healthy control group), with the highest levels observed in the severe CHB group († *p* < 0.05 vs. non-severe CHB group). Statistically significant differences were found for ALT, AST, PLT, and bilirubin levels between groups. Statistical significance: ^*^p < 0.05, ^**^p < 0.01

### ROC curve and clinical applicability

**Fig. 2:**
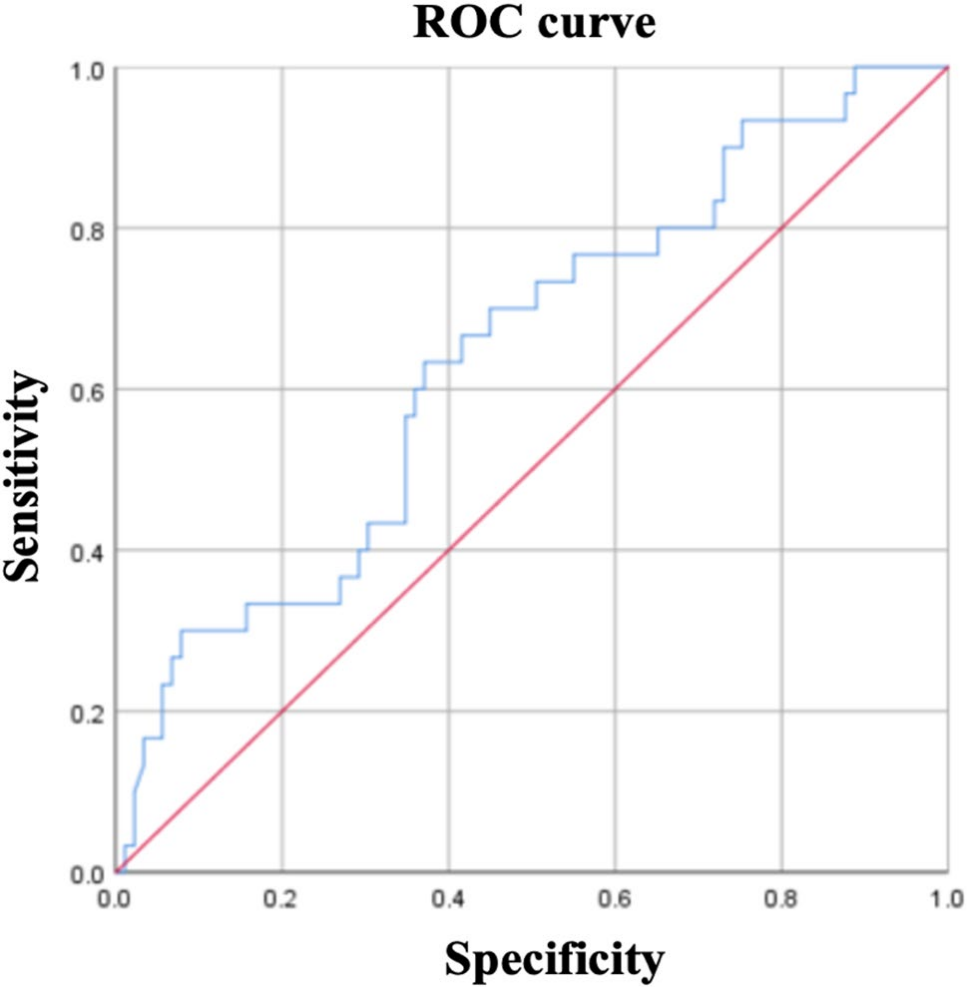
Receiver operating characteristic (ROC) curve for serum TIM4 levels in discriminating severe from non-severe CHB patients. The area under the curve (AUC) was 0.640 (95% CI: 0.527–0.754, *p* = 0.022), indicating a moderate ability to predict severe CHB. The optimal TIM4 cutoff value of 447.68 pg/mL was determined by maximizing Youden’s index, providing a sensitivity of 63.3% and specificity of 62.9%

ROC curve analysis was performed to evaluate the diagnostic utility of serum TIM4 for distinguishing.

severe from non-severe CHB **(**Fig. 2**)**. The area under the ROC curve (AUC) was 0.640 (95% CI:

0.527–0.754, *p* = 0.022). The optimal TIM4 cutoff value was 447.68 pg/mL, providing a sensitivity of 63.3% and specificity of 62.9%.

### Correlation between serum TIM4 and laboratory markers

Bivariate correlation analysis showed significant associations between TIM4 and key markers of liver injury (Table 3). TIM4 exhibited positive correlations with ALT (*r* = 0.33, *p* < 0.01), AST (*r* = 0.35, *p* < 0.01), and bilirubin levels (TBIL (*r* = 0.16, *p* = 0.04) and DBIL (*r* = 0.29, *p* < 0.01)), all of which are indicators of liver damage. A strong negative correlation was observed between TIM4 and PLT (*r* = -0.36, *p* < 0.01), which is commonly associated with portal hypertension and advanced liver disease.

These findings suggest that elevated TIM4 levels are closely related to liver dysfunction and could potentially reflect the degree of immune suppression and inflammation in the liver microenvironment, both of which are central to the pathogenesis of severe CHB. Notably, the inverse correlation between TIM4 and PLT underscores its relevance as a marker for thrombocytopenia, a well-known consequence of liver cirrhosis and portal hypertension. No significant correlations were found between TIM4 and WBC, RBC, Hb, BUN, or SCr.

**Table 3:** Correlation analysis between serum TIM4 levels and laboratory parameters

Parameter	Correlation Coefficient (*r*)	*p*-value
ALT	0.33	< 0.01^**^
AST	0.35	< 0.01^**^
TBIL	0.16	0.04
DBIL	0.29	< 0.01^**^
WBC	-0.08	0.31
RBC	-0.07	0.38
Hb	-0.03	0.73
PLT	-0.36	< 0.01^**^
BUN	-0.04	0.65
SCr	0.02	0.80

TIM4 levels with laboratory parameters. TIM4 showed significant positive correlations with ALT, AST, and bilirubin levels (all *p* < 0.05), and a negative correlation with PLT (*p* < 0.01). No significant correlations were found between TIM4 and WBC, RBC, Hb, BUN, or SCr. ^**^p < 0.01

### Identification of TIM4 as a risk factor for severe CHB

Univariate binary logistic regression was performed to evaluate factors associated with progression to severe CHB (Table 4). Among all laboratory parameters analyzed in univariate models, serum TIM4 level emerged as a significant independent risk factor (OR = 1.005, 95% CI: 1.001–1.009, *p* = 0.022).

In this model, traditional markers like ALT, AST, and PLT did not reach statistical significance as independent predictors of severity.

**Table 4:** Univariate binary logistic regression analysis for factors associated with severe CHB

Variable	Coefficient	S. E	Wald Value	df	*p*-value	OR	95% CI for OR
TIM4 (pg/mL)	0.005	0.002	5.222	1	0.022^*^	1.005	1.001–1.009
ALT	0.000	0.001	0.135	1	0.713	1.000	0.999–1.001
AST	0.000	0.001	0.398	1	0.528	1.000	0.999–1.002
TBIL	0.003	0.002	3.298	1	0.069	1.003	1.000-1.006
DBIL	0.003	0.002	2.342	1	0.126	1.003	0.999–1.008
PLT	-0.004	0.003	1.472	1	0.225	0.996	0.990–1.002

S. E., Standard Error; df, degree of freedom; OR, Odds Ratio; CI, Confidence Interval. ^*^p < 0.05

To further evaluate the independence of TIM4 from key demographic confounders, a multivariate binary logistic regression model was performed, adjusting for age and sex. As shown in Table 5, serum TIM4 remained a statistically significant independent predictor of severe CHB (adjusted OR = 1.005, 95% CI: 1.001–1.009, *p* = 0.028) after adjustment. Age was also a significant independent predictor in this model (adjusted OR = 1.052, *p* = 0.004), while sex was not (*p* = 0.380).

**Table 5:** Multivariate binary logistic regression analysis for factors associated with severe CHB

Variable	Coefficient	S. E	Wald Value	df	*p*-value	aOR	95% CI for OR
TIM4 (pg/mL)	0.005	0.002	4.822	1	0.028 ^*^	1.005	1.001–1.009
Age (year)	0.051	0.018	8.342	1	0.004 ^**^	1.052	1.016–1.089
Sex (Male)	0.512	0.584	0.769	1	0.380	1.669	0.531–5.240

S. E., Standard Error; df, degree of freedom; OR, Odds Ratio; CI, Confidence Interval. The model included TIM4, age, and sex (reference: female) as covariates. ^*^p < 0.05. ^**^p < 0.01

### Predictive performance and clinical applicability of serum TIM4 for severe CHB

The diagnostic utility of serum TIM4 for predicting severe CHB was evaluated using ROC curve analysis (Fig. 2; Table 6). The area under the ROC curve (AUC) was 0.640 (95% CI: 0.527–0.754, *p* = 0.022), indicating a statistically significant but modest predictive ability. The optimal cutoff value for TIM4, determined by maximizing Youden’s index, was 447.68 pg/mL, which provided a sensitivity of 63.3% and a specificity of 62.9%.

To evaluate the comparative predictive performance of serum TIM4 and conventional liver enzymes, ROC analyses were conducted for TIM4, ALT, and AST. The AUC for ALT was 0.620 (95% CI: 0.505–0.735), for AST was 0.635 (95% CI: 0.522–0.748), and for TIM4 was 0.640 (95% CI: 0.527–0.754). While TIM4 showed a numerically slightly higher AUC, the overlapping CI indicates that its discriminatory ability for severe CHB is comparable to, but not statistically superior to, that of traditional liver enzymes (Table 6).

To investigate whether a multi-marker approach could improve prediction, a logistic regression model combining TIM4, ALT, and AST was developed. The ROC curve analysis based on the predicted probabilities from this model yielded an AUC of 0.685 (95% CI: 0.578–0.792), with a sensitivity of 66.7% and a specificity of 65.2% probability threshold (Youden’s index) (Table 6).

For combined biomarker analysis, a multivariable logistic regression model including TIM4, ALT, and AST was constructed, and predicted probabilities from this model were used to generate the ROC curve.

**Table 6:** ROC curve analysis of TIM4 for predicting severe CHB

Biomarker	Optimal Cutoff	AUC (95% CI)	*p*-value	Sensitivity	Specificity
TIM4	447.68 pg/mL	0.640 (0.527–0.754)	0.022^*^	63.3%	62.9%
ALT	—	0.620 (0.505–0.735)	—	—	—
AST	—	0.635 (0.522–0.748)	—	—	—
TIM4 + ALT + AST	—	0.685 (0.578–0.792)	—	66.7%	65.2%

The optimal cutoff was determined by maximizing Youden’s index. A TIM4 level ≥ 447.68 pg/mL may serve as a potential threshold for identifying CHB patients at elevated risk for severe disease progression. For the combined model, ROC analysis was performed using predicted probabilities derived from a multivariable logistic regression model incorporating TIM4, ALT, and AST. * *p* < 0.05

Crucially, the cutoff value of 447.68 pg/mL and its associated performance metrics are derived from this single-center cohort. External validation in independent, multi-center populations is essential before this threshold can be considered for clinical application.

## Discussion

This study offers new clinical evidence that serum levels of the immunoregulatory molecule TIM4 are significantly higher in patients with CHB and are positively linked to disease severity. Our main findings show that (1) TIM4 levels rise progressively from healthy controls to non-severe and then to severe CHB; (2) TIM4 is associated with traditional markers of liver injury and inversely related to PLT count, a marker connected to portal hypertension and advanced disease; (3) TIM4 is an independent risk factor for severe CHB; and (4) TIM4 has a moderate yet statistically significant ability to predict which patients are at risk of severe disease progression.

The observed stepwise increase in serum TIM4 from healthy controls to non-severe and severe CHB patients may reflect the dynamic immune landscape characteristic of CHB as an immune-mediated disease. The ongoing exposure to HBV antigens in CHB is understood to foster an immunosuppressive hepatic microenvironment, which is associated with T-cell exhaustion [21, 22]. TIM4, expressed on antigen-presenting cells like macrophages, is known to deliver co-inhibitory signals to activated T cells via TIM1, thereby potentially modulating their activity [8–10]. Consequently, the elevated TIM4 levels observed in our CHB cohort, especially those with severe cases, could be indicative of an enhanced immunoregulatory or immunosuppressive response, which may be an adaptive response to reduce sustained hepatic inflammation. This association suggests a potential role for elevated TIM4 as a marker of an altered immune environment that could be permissive for disease progression. Our findings are in line with prior studies reporting dysregulated TIM4 expression in other settings of chronic inflammation and liver fibrosis, supporting its relevance in immune-mediated liver pathology [8, 23]. However, the cross-sectional and associative nature of our study precludes definitive causal inferences. Whether elevated TIM4 directly drives immune suppression and liver injury in CHB, or primarily serves as a bystander marker of the underlying immunosuppressive environment, remains a critical question for future mechanistic investigations.

The significant correlations between TIM4 and markers of hepatic injury (e.g., ALT, AST) and function (e.g., bilirubin) reinforce its potential role in the inflammatory cascade of CHB. More notably, the strong negative correlation with PLT count is intriguing. Thrombocytopenia in advanced CHB is often a consequence of portal hypertension. This association suggests that elevated TIM4 may be linked not only to inflammatory activity but also to the hemodynamic and fibrotic sequelae of chronic liver disease. This aligns with recent research implicating TIM4 in macrophage-mediated processes that can influence tissue fibrosis [8].

The most significant contribution of this study is the identification of TIM4 as an independent risk factor for severe CHB via logistic regression. This was initially suggested by univariate logistic regression and was robustly confirmed in a multivariate model adjusting for age and sex (aOR = 1.005, *p* = 0.028; Table 5). While traditional liver enzymes did not retain independent predictive value in our model, TIM4 emerged as a significant predictor. This suggests that TIM4 may capture a distinct dimension of disease pathophysiology, namely, the immune dysregulation state, that complements standard biochemical markers.

The subsequent ROC analysis demonstrated that TIM4 possesses a modest yet statistically significant predictive value (AUC = 0.640) for identifying severe CHB. This level of discriminatory power indicates that TIM4 alone is unlikely to serve as a standalone diagnostic biomarker. However, its true clinical potential may lie in functioning as a complementary component within a multimodal risk stratification panel. Integrating TIM4 levels with established and routinely measured markers of liver injury (e.g., ALT, AST) and portal hypertension (e.g., PLT) could enhance the overall accuracy of identifying patients at high risk of disease progression, thereby enabling more informed and personalized clinical decision-making.

Although TIM4 alone demonstrated only modest discriminatory ability, the combined model incorporating TIM4 with ALT and AST achieved a higher AUC (0.685), suggesting that TIM4 may provide complementary rather than redundant information to routine liver enzymes and may be more useful as part of a multi-marker risk stratification approach. However, the improvement was modest, underscoring that TIM4 is best viewed as a supplementary biomarker within a broader clinical assessment. Furthermore, it is imperative to emphasize that the optimal cutoff value identified in this study (447.68 pg/mL) and its associated sensitivity and specificity are intrinsically tied to our specific cohort and methodological context. As a finding from a single-center, cross-sectional study, this cutoff requires rigorous external validation in independent, prospective, and preferably multi-center cohorts before any translation to clinical practice can be responsibly suggested. Future studies should also explore dynamic changes in TIM4 levels over time and in response to therapy, which could provide further insights into its utility as a monitoring tool.

Our findings should be interpreted considering certain limitations. First, the cross-sectional design precludes the assessment of causality or temporal changes. Longitudinal studies are needed to determine if TIM4 levels predict future disease progression. While our results show a significant association between TIM4 levels and disease severity, we cannot determine whether elevated TIM4 directly contributes to CHB progression or is a consequence of advanced disease. External validation cohorts and longitudinal follow-up studies are required before TIM4 can be adopted as a standalone predictive biomarker in clinical practice. Additionally, the single-center nature of our study may limit the generalizability of our findings to other populations or healthcare settings. Second, the sample size, especially in the severe CHB group, was limited, and larger and multi-center cohorts are needed to confirm the robustness and generalizability of these findings. Furthermore, body mass index (BMI) and detailed comorbidity data were not systematically available for all participants and, therefore, could not be included in the baseline analysis. Future studies should incorporate these variables to better control for metabolic and systemic factors that may influence TIM4 expression and disease severity. Third, in this study, potential confounding factors that could influence TIM4 levels (e.g., metabolic or autoimmune comorbidities) were not fully addressed, and future research should control for these variables. Fourth, while we discuss the potential immunoregulatory role of TIM4 based on existing literature, our study design does not provide functional evidence. Therefore, the observed associations should be interpreted as correlative, and causal relationships require validation through future mechanistic studies. Finally, mechanistic studies are necessary to understand whether TIM4 has a direct role in causing liver injury or serves primarily as a marker of the immunosuppressive environment in CHB. Exploring the cellular sources of soluble TIM4 in CHB and its effects on HBV-specific T cells in in vitro and in vivo models would offer valuable insights into its role in disease development.

Identifying TIM4 as a predictive biomarker could significantly improve risk assessment in CHB patients by enabling earlier identification of individuals at higher risk of disease progression. This may support more personalized monitoring strategies and earlier interventions. Beyond its diagnostic potential, TIM4 may also play an important role in monitoring disease progression over time. Given that CHB often follows fluctuating courses of exacerbation and remission [24], tracking TIM4 longitudinally may help detect early transition toward severe disease, including cirrhosis or HCC, and guide follow-up intensity and surveillance strategies.

## Conclusion

Overall, this study demonstrates that elevated serum TIM4 is associated with increased disease severity in CHB and serves as an independent risk factor for severe progression. Although TIM4 alone is not sufficient as a standalone diagnostic biomarker (AUC = 0.640), it may provide a complementary value when combined with established clinical parameters. These findings suggest that TIM4 may provide valuable insights into the immunopathological mechanisms underlying CHB progression and could be integrated into clinical practice to help tailor monitoring and intervention strategies for individual patients.

Future studies are needed to validate these findings in larger, prospective, and multi-center cohorts, and to further explore the clinical utility of TIM4 in combination with other biomarkers, as well as its potential role as a therapeutic target in CHB management.

## Data Availability

The data that support the findings of this study are available on request from the corresponding author. The data are not publicly available due to privacy or ethical restrictions.

## Abbreviations

CHB: Chronic Hepatitis B
TIM4: T-cell Immunoglobulin and Mucin Domain-containing Protein 4
HBV: Hepatitis B Virus
ALT: Alanine Aminotransferase
AST: Aspartate Aminotransferase
PLT: Platelet Count
ROC: Receiver Operating Characteristic
AUC: Area Under the Curve
ELISA: Enzyme-Linked Immunosorbent Assay
TBIL: Total Bilirubin
DBIL: Direct Bilirubin
WBC: White Blood Cell Count
RBC: Red Blood Cell Count
HBcAb-IgM: Hepatitis B Core Antibody Immunoglobulin M
ANOVA: Analysis of Variance
LSD: Least Significant Difference (post-hoc test)
CI: Confidence Interval
OR: Odds Ratio
SD: Standard Deviation
IQR: Interquartile Range
BMI: Body Mass Index
BUN: Blood Urea Nitrogen
SCr: Serum Creatinine
Kruskal-Wallis H test: A non-parametric test for comparing more than two independent samples
Mann-Whitney U test: A non-parametric test for comparing two independent samples
